# Re-imagining approaches for mental health and substance use health workforce regulation in Canada: Making room for dynamic tensions

**DOI:** 10.1371/journal.pmen.0000168

**Published:** 2025-03-10

**Authors:** Micheala Slipp, Sophia Myles, Jelena Atanackovic, Gordon Garner, Mary Bartram, Ivy Lynn Bourgeault, Kathleen Leslie

**Affiliations:** 1 Faculty of Health Disciplines, Athabasca University, Athabasca, Alberta, Canada; 2 School of Sociological and Anthropological Studies, University of Ottawa, Ottawa, Ontario, Canada; 3 Laurentian University, Sudbury, Ontario, Canada; 4 Systemic Consulting Mental and Substance Use Health (SCMSH Ltd.), Ottawa, Ontario, Canada; 5 Mental Health Commission of Canada, Ottawa, Ontario, Canada; 6 School of Public Policy and Administration, Carleton University, Ottawa, Ontario, Canada; PLOS: Public Library of Science, UNITED KINGDOM OF GREAT BRITAIN AND NORTHERN IRELAND

## Abstract

Regulation of the mental health and substance use health (MHSUH) workforce affects not only quality but also the availability and accessibility of services. This study explores the perspectives of subject matter experts on how flexible, modern regulatory reforms in the MHSUH workforce can promote more equitable access to services and the impact of regulation on service safety, quality, accessibility, and the workforce. Between January and March 2023, semi-structured interviews were conducted with 15 subject matter experts from diverse MHSUH provider groups and organizations. Participants shared insights on regulation, quality assurance, and relevant policies that shape equitable access to MHSUH services and workforce dynamics. The data were analyzed thematically, and findings were validated by the project’s diverse advisory committee. The analysis generated four themes that underscore the need to re-imagine regulatory approaches in a way that makes room for the dynamic tension between standardization and protection of the public on the one hand, and flexibility and respect for the value of lived expertise on the other. These themes include: (1) the impact of policy exclusion, criminalization, and stigma of MHSUH; (2) gaps in regulation limit access and the protection of the public; (3) the dynamic tension between standardization and flexibility in regulatory approaches; and (4) policies to support the integration of lived experience into the MHSUH and broader health workforce are limited. In conclusion, re-imagined regulatory approaches are essential for ensuring equitable access to MHSUH services and addressing significant gaps in workforce planning. An adapted right-touch regulation approach, which aligns regulatory interventions with the level of risk, may help guide future reforms for the MHSUH workforce in Canada. This approach should balance the need to appropriately manage risks of harm while avoiding unnecessary barriers to practice across the spectrum of MHSUH providers.

## Introduction

The mental health and substance use health (MHSUH) workforce is in critical demand [1], not least because of the widespread MHSUH challenges and needs exacerbated during the COVID-19 pandemic [2–8]. The pandemic heightened mental health concerns, social isolation, and financial stress while fuelling the pre-existing overdose and toxic supply crises [9–11], all of which can have serious and long-lasting MHSUH impacts [6,12]. These pandemic impacts have equity implications because they disproportionately affect key groups with higher risk factors, including women with younger children, people who live alone, those with a previous diagnosis of a mental health or substance use disorder, people who identify as 2SLGBTQ+, people who have a low income or are unemployed, and Indigenous peoples [7,13,14]. Adolescents and young adults with a history of adverse childhood experiences were also more likely to exhibit pandemic-related symptoms and stressors [15]. Access to virtual service delivery mitigated some of these impacts but remains highly variable and inequitable across the country in the absence of national service standards [16,17].

Key to addressing the growing gap in meeting service needs is the availability and accessibility of qualified MHSUH providers [18]. While the long-term impacts of the pandemic are still uncertain [12], there is evidence that MHSUH service needs are not being met. According to the latest data from Statistics Canada [19], half of the people who need MHSUH services have not talked to a health professional about their mental health in the past year. Almost two-thirds of people in Canada recently gave mental health services a failing grade regarding access, public confidence, satisfaction and effectiveness and 60% gave substance use health services a similarly poor grade [18].

A key factor impacting MHSUH workforce and service availability, accessibility, and quality is the effectiveness of workforce regulatory frameworks [20]. Regulatory frameworks can impact decisions regarding funding for services, including employment-based benefit plans, public health insurance, and direct public funding, affecting access to health care providers and their services [21]. In the broader health workforce literature, statutory regulation has been connected to optimizing the workforce [22–25]. Right-touch or risk-based regulatory frameworks have also gained momentum during the COVID-19 pandemic, focusing on matching the strength of regulation with the level of risk to the public in a way that does not create unnecessary barriers to practice [26–30]. For example, cross-jurisdictional licensing and registration requirements were loosened and scopes of practice of regulated providers in hospital settings were expanded as part of pandemic response measures [31,32].

In Canada, regulatory modernization efforts for the MHSUH workforce vary across the country and include, for example, statutory changes to regulation of counselling therapy in Nova Scotia [33], New Brunswick [34], and Prince Edward Island [35], and to psychotherapy in Ontario [36]. Alberta and British Columbia have advanced towards statutory regulation of counselling therapy and psychotherapy in 2024 [37]. Voluntary certification programs for the peer support and addiction counselling workforces have been developed in Canada, along with competency frameworks for the substance use health and psychosocial rehabilitation workforces [38–42].

This variable regulatory landscape for MHSUH providers in Canada can negatively impact equitable access to care and workforce mobilization. The inconsistencies in statutory regulatory frameworks, especially for virtual care, include variable practice standards for documentation and privacy [43,44]; confusing and uncoordinated reciprocal licensure and liability insurance requirements [45,46]; and new licensure categories specific to virtual care that only apply to certain MHSUH providers in certain provinces [47]. In addition to improving the availability and accessibility of MHSUH service providers regulated by statute, international literature identifies a need to improve the visibility, integration, and support for MHSUH providers that are not formally regulated [1].

Finding the appropriate regulatory framework that protects the public without undue barriers to practice for different MHSUH provider groups is a complex endeavour. Currently, occupational regulation in the MHSUH workforce exists on a spectrum from voluntary to statutory. While regulation is often narrowly associated with statutory regulation, for this study, we define regulation more broadly as the legal or occupational rules governing entry, membership, and conduct within these MHSUH service provider groups [48]. Within this broad definition, the main regulatory frameworks include voluntary certification, co-regulation, and occupational licensing models [49]. In voluntary certification, members join an association and agree to abide by rules of conduct and codes of ethics. The association may publish a register of members and operate a complaints mechanism, but the system is voluntary and not underpinned by statute [49]. While voluntary certification meets the broad definition of regulation used in this paper, some certification bodies may explicitly reject the notion of regulation, given the association with top-down government statutes. Co-regulatory models are similar to voluntary certification, but the certification functions offered by provider associations are delegated or recognized by government (for example, as an eligibility criterion for public funding). Occupational licensing or statutory registration systems are the most prescribed, referring to regulatory schemes with a regulatory body established through legislation and often including protected titles and scopes of practice. These various regulatory frameworks serve to mitigate risk through various functions, generally divided into *ex ante* functions (directed at entry to practice competencies and requirements, which may include specific education or academic credentials) and *ex post* functions (focused on addressing competence and conduct after entering practice, such as maintaining certification, setting standards, monitoring competence, and addressing complaints about providers) [50].

In Canada, there are wide variations in the regulation of the MHSUH workforce along this spectrum. Some provincial or territorial licensing models cover physicians, psychologists, social workers, and occupational therapists across the country, leaving uneven statutory registration of psychotherapists or counselling therapists and psychiatric nurses, and reliance (in part as a matter of preference, in part as a result of policy neglect) on voluntary certification models and competency frameworks for addictions counsellors, harm reduction workers, peer support workers, and psychosocial rehabilitation workers [38,39,51–54]. These regulatory differences complicate the provision of care, whether in person or virtually delivered [45,55], highlighting the critical need for flexible and nuanced frameworks across the country to support equitable access to MHSUH services. Such regulatory frameworks seem to be necessary to support growing trends in MHSUH reform such as provider diversification, appreciation for the role of the peer support workforce, more flexible scopes of practice, decriminalization, stigma reduction, integration and person-centred care, and parity with physical health [56]. These changes are all occurring in the context of a MHSUH system that has long faced gaps and inequities in public insurance coverage under Canadian Medicare [1,24,57,58].

This study gathered perspectives from a range of subject matter experts on two focus areas: (1) how regulation and potential regulatory reform impact the safety and quality of services, access to services, and the workforce; and (2) how flexible, modern regulatory reforms in the MHSUH workforce can facilitate more equitable access to MHSUH services. Our research was guided by our pan-Canadian advisory committee composed of leaders and subject matter experts from different sectors related to the MHSUH workforce, including those with lived and living experience. This research builds on our recent scoping review on regulating the MHSUH workforce, which aimed to identify promising practices for regulatory reform in this sector [1].

## Methods

This study used an exploratory qualitative design [59]. We conducted interviews with subject matter experts recruited in consultation with our pan-Canadian advisory committee. We followed the Consolidated Criteria for Reporting Qualitative Research (COREQ) [60] to guide the reporting of our methods and findings (see S1 Table, COREQ Checklist).

### Data collection

We used purposive sampling [61] to identify subject matter experts with particular knowledge about MHSUH regulation and quality assurance at the national and provincial/territorial levels across the country. Twenty potential participants were contacted by email with information about the study and invited to participate in semi-structured interviews. Before the interview, written consent was obtained from each of the 15 individuals who agreed to participate. Five invitations were declined; however, these potential participants did not significantly differ from those who did respond. Many respondents represented pan-Canadian organizations and were familiar with regulated and non-regulated occupations in all Canadian provinces and territories. While nuanced local contexts are important, the views offered covered sufficiently broad perspectives to enhance the trustworthiness and transferability of the results [62]. Interviewees represented various occupations and provided insights on both mental health and substance use health. The perspectives that participants identified with included mental health provider (n = 7), substance use health provider (n = 6), peer/lived experience provider (n = 7), professional provider (n = 5), and regulatory/certification/association representative (n = 5). Since some participants held multiple roles and offered perspectives from different angles, the total number of perspectives exceeds the number of individual participants. For example, some interviewees identified with dual roles as substance use health providers and lived experience providers, while others identified with roles as professionals in both the mental health and substance use health sectors. Twelve respondents represented nationally scoped organizations that interface with all regulated and non-regulated jurisdictions.

Two research team members were present for all interviews: one to facilitate (KL or MB) and another to take notes and ask follow-up probing questions (MS, JA, SM). At participants’ request or as schedules permitted, interviews included one or two individuals representing a particular organizational, policy, or provider group perspective. Between 1 January 2023 and 31 March 2023, we conducted 14 interviews with the 15 participants via videoconference on Microsoft Teams; one interview included two participants. Interviews lasted approximately 60 minutes and were recorded and transcribed verbatim.

The interview guide was also informed by evidence from a previous scoping review of Canadian and international literature on regulation and quality assurance for the MHSUH workforce [1] and was distributed to participants before their interviews (see S2 Table, Semi-Structured Interview Guide). Broadly, we sought perspectives on the impact of regulation and regulatory reform on the safety and quality of MHSUH services, as well as access to services and providers. Participants were asked questions about current regulatory or quality assurance mechanisms; the impact of regulation on the public, providers, and practice; desired changes or knowledge to help ensure equitable access to safe and high-quality MHSUH services, as well as anything else they wished to tell us about regulation and quality assurance for the MHSUH workforce in Canada.

### Data analysis

The data were analyzed thematically [63] using NVivo qualitative analysis software [64]. This project used template thematic analysis [65], a form of codebook thematic analysis [66] that uses a set of *a priori* codes derived from research questions and an initial review of a subset of transcripts conducted by several researchers. Reflections and impressions of the wider research team were considered when developing the initial template. Moving inductively throughout the entire dataset, the primary data analyst (MS) developed the initial codebook following multiple readings of the transcripts. The codebook was iteratively revised based on discussions with the research team. Initial themes were developed based on a review of the coded and original text by MS and subsequently refined through research team discussions. The initial thematic analysis was verified with the project advisory committee. Research team deliberations, deep engagement with the transcripts, and advisory committee feedback enhanced the trustworthiness of the analysis.

### Ethical considerations

Approval for this study was provided by Athabasca University (File No. 24761) and the University of Ottawa (File No. S-05-22-8126) research ethics boards. Informed consent was obtained in writing and verbally from all interview participants. No participants withdrew from the study. Participants were assigned a unique code (represented by P1 through P15). Quotations presented in the findings are attributed to these participant identification codes and the provider or organizational perspective they represent without using position titles. Further contextual information cannot be disclosed due to ethical requirements to prevent potential identification.

### Research team positionality

This interdisciplinary team includes six women and one man, all researchers at different career stages and with backgrounds in sociology, regulation, political science, health policy, nursing and law. Three authors identify as members of the MHSUH workforce, one as a clinical supervisor, counsellor, and art therapist with a focus on trauma-informed therapy (MS), one as a social worker (MB), and one as a systemic stigma analyst currently working as an innovation and transformation specialist and with extensive personal experience seeking and accessing MHSUH services with a trauma-informed life (GG).

## Results

We constructed four themes from the data: (1) the impact of policy exclusion, criminalization, and stigma; (2) gaps in regulation limit access and public protection; (3) the dynamic tension between standardization and flexibility; and (4) policies to support the integration of lived experience into the MHSUH and broader health workforce. These four themes underscore the need to re-imagine regulatory approaches for the MHSUH workforce in Canada in a way that makes room for the dynamic tension between standardization and protection of the public on the one hand, and flexibility and respect for the value of lived expertise on the other. A summary of themes and illustrative quotes are provided in Table 1.

**Table 1:** Themes and illustrative quotes.

Theme	Description	Illustrative Quotes
Impact of policy exclusion, criminalization, and stigma	Respondents highlighted how stigma has led to the exclusion of MHSUH issues from important policy and funding initiatives and developments. This stigma has been compounded by a history of criminalization of substances.	“[There is] gross underfunding of mental health and substance use services to begin with.” “So because it’s a criminal substance, there are people who have had to fill the…gaps in services…without any regulation.”
Gaps in regulation limit access and public protection	Respondents discussed how gaps in access to MHSUH services stem from varied regulation of the workforce across jurisdictions. Regulatory status impacts service availability (including cross-jurisdictional virtual services), insurance eligibility, and access to team-based information, excluding many MHSUH workers who are not statutorily regulated.	“[Addiction counselling is] an unregulated profession, therefore, not covered by any insurance in the country, which means every service of ours is self-pay unless you fall under a treatment facility or a doctor.” “So the fact that there’s different regulation [for psychotherapy or counselling therapy] doesn’t impact [insurers] as much, but the fact that there is no regulation in some provinces does.”
Dynamic tension between standardization and flexibility	Respondents identified tension around the issue of standardizing regulation across the MHSUH spectrum of workers. While all respondents agreed on the importance of quality assurance for MHSUH services, there was a divide between those respondents who focused on the need for greater standardization and harmonization, and those who expressed concerns about the risks of over-regulation, particularly for peer support workers and others who draw on lived experience.	“There needs to be enforceable standards…People are too vulnerable.” “Credentialism is a capitalistic sort of thing. And peer support is not intended to be rooted in capitalism.”
Supporting the integration of lived experience	Respondents discussed workforce policies that constrain the pay, advancement, and overall integration into the workforce of MHSUH service providers who are not regulated by statute. Many of these providers draw on lived experience to fill vital roles, particularly in underserved communities, but face challenges fitting within traditional regulatory structures that discourage self-disclosure.	“Experience, which is ironically, exactly what you’re looking for, turns out to be the barrier that stops you from being able to get a job.” “If we don’t have [peers] involved, and we don’t have them in the right way, that [substance use health] continuum will collapse.”

### Impact of policy exclusion, criminalization, and stigma

Interviewees noted the significant impacts of policy exclusion in shaping how MHSUH services are organized and delivered across Canada. One participant commented that *“…[there is] gross underfunding of mental health and substance use services to begin with” [P14].* Participants identified a siloed system with separate buildings, lines, and funding, and discussed how stigma is *“rooted in the historic separation of addiction treatment and financing from mainstream health care” [P6]*.

Policy legacies arising from the exclusion of MHSUH from Medicare and the criminalization of substances have influenced regulation of the MHSUH workforce, and these factors have been compounded by stigma. Participants discussed how unconscionable it would be, for example, to think about verifying an orthopedic surgeon’s competence to set a leg, yet *“...in mental health? We have to because we don’t know” [P8].* However, they predominantly discussed how substance use is stigmatized and politicized and focused on addictions and criminalization rather than the more positive conceptualization of substance use health. As one participant noted, “*stigma and the unconscious bias around addiction…is driving a lot of policy decisions. And it’s leading to poor outcomes for people and poor service provision. And in sometimes unsafe situations, very unsafe situations for vulnerable people” [P1].* The politicization of substance use was viewed to impact how governments or jurisdictions “*view the people who are desiring… or needing of health service and what kind of health service they may deserve, and what kind of safety protections they may deserve” [P1].* Interviewees working in substance use health also identified a layer of mistrust in government given the history of criminalization: *“...So because it’s a criminal substance, there are people who have had to fill the … gaps in services…without any regulation” [P9].* These policy gaps related to stigma and criminalization resulted in service gaps, limiting access to MHSUH services and providers.

### Gaps in regulation limit access and public protection

#### Impacts on insurance coverage and inclusive, accessible services.

Regulatory gaps limit access to the full range of MHSUH supports. Some participants identified how statutory regulation acts as a gatekeeper to care from a risk management perspective. Insurance coverage in particular was seen as facilitating access to providers who are regulated by statute and limiting access to providers who are regulated through voluntary certification, “*Most insurance plans will only reimburse regulated health care providers. And so you’ve got your standard psychologists, masters of social work. Pretty much that’s it” [P10].* Participants reflected on the barriers this creates to accessing substance use health treatment:

*So one of the things to keep in mind is [addiction counselling is] an unregulated profession, therefore, not covered by any insurance in the country, which means every service of ours is self-pay unless you fall under a treatment facility or a doctor…[P3].*

Eligibility requirements for federally-funded mental health counselling also limit access for First Nations and Inuit, who “*could only claim for services if you had a regulated provider” [P8].*

To create inclusive, accessible services, participants proposed that regulation and quality assurance mechanisms be strength-based, trauma-informed, recovery-oriented, sustainable, and culturally safe: “*Just the importance of recognizing different intersectionalities and … how much of substance use problems are driven in trauma … would obviously be critical to any sort of regulations or accountability frameworks” [P9].* Further, participants spoke to strengthening access to care that honours First Nations, Inuit and Métis wisdom, as well as the importance of collaborating with local Indigenous communities in developing practice and governance standards for MHSUH care, “*We can’t impose standards on the Indigenous peoples, because … they have a different certification, they have a different entryway to practice, and they have different practice modalities” [P3].*

#### Access to health data is impacted by regulatory status of providers.

With respect to access and information sharing among providers, some participants noted that legislative frameworks limit the circle of care to the point of excluding unregulated providers from health care teams, “*Because they’re not regulated professions, they are not given access to the same information that others are” [P14].* The requirements of privacy legislation, which limit information sharing in care teams, may leave other providers, like peer support workers and counsellors, out of care planning. Limiting the involvement of providers who can participate in care planning reduces opportunities for holistic approaches to care that treat the whole person rather than the illness.

#### Jurisdictional differences and capacity issues impact access to care.

Inconsistencies in provincial and territorial statutory regulation of psychotherapy and counselling therapy also impede access to MHSUH care, *“So the fact that there’s different regulation doesn’t impact [insurers] as much, but the fact that there is no regulation in some provinces does” [P10].* Establishing a pan-Canadian regulatory body that could provide access to a broader group of therapists across the country was suggested as a way to support cross-jurisdictional virtual service provision. Participants also identified the role that insurance coverage plays in constraining access to the full range of providers, *“Insurers…will cover people who are regulated…if my plan has very good coverage of several thousand a year, then I can’t afford to not use that and to try to find help elsewhere…access is so limited by regulation in that way” [P11].* Further, at the time of data collection, “*Counselling therapists and psychotherapists [were] the only mental health group that have to charge GST and HST” [P8].*

These gaps and barriers in access are particularly problematic given how capacity issues have been exacerbated during the pandemic. Regarding substance use health, one participant observed:

*The workforce is depleted, right? Like we’re absolutely disheveled and depleted right now because of COVID...Because we don’t have the workforce, we don’t have the right amount of people. And the people we did have are burnt out or they’re retiring. And so we just are in a very precarious situation from a workforce perspective [P3].*

A similar sentiment was expressed about private sector services offered by counselling therapists and psychotherapists, “*In Ontario, we found out that prior to COVID, most counsellors had room in their practice to bring on new clients… continuously. They don’t now, they’re chock full” [P8]*. Finding ways to improve access to services and providers in a safe way was a focus for many respondents. Participants broadly agreed that gaps in regulation impacted access to care and acknowledged that finding the appropriate regulatory framework across the spectrum of MHSUH providers was a complex endeavour.

### Dynamic tension between standardization and flexibility

A striking dynamic tension between standardization and flexibility permeated the interviews. There was a divergence of perspectives on whether statutory regulation was necessary for all segments of the MHSUH workforce, the form that regulation should take for different provider groups, and the value of harmonizing regulation across jurisdictions and provider groups. While all participants agreed on the importance of quality assurance for MHSUH services, some focused more on the need for greater standardization and harmonization, while others focused more on the need for flexibility to avoid overly strict regulation, particularly for peer support workers and those who draw on lived experience

#### Cross jurisdictional harmonization.

Some participants stressed the importance of consistency and clarity through harmonized standards for MHSUH service provision across the country, emphasizing the confusion that interjurisdictional variation creates for providers and the public. One participant described conversations about legislation and service delivery in Canada with international peers who noted it is like “*you have ten different countries” [P2]*. Participants pointed out that inconsistent standards lead to confusion and uncertainty in virtual care across different regions: *“One of the biggest issues that happened - and this is COVID driven - is that when everybody went online, it actually opened up the opportunity to serve clients not in your location…Nobody actually understands the rules…it was just really mucky.” [P8]*.

In addition to permitted service modalities by providers in different locations, variations in the use of protected titles between provinces were another highlighted issue. For example, in New Brunswick, Nova Scotia, and Prince Edward Island, ‘counselling therapist’ is a regulated title, while in Ontario and Quebec, ‘psychotherapist’ is regulated, along with its scope of practice. Some participants raised public safety concerns that emerge with these jurisdictional regulatory inconsistencies and a resultant lack of accountability. One participant suggested that “*to make a strong system of health, it’s about a system of health and a system of accountability. The more we can harmonize those standards across the country, the better off we will be*” *[P1]*.

A lack of accountability regarding quality of substance use health services was also discussed, *“…If you’re accessing a private facility, you have actually no sort of guarantees about what kind of services you may receive” [P9].* Additionally, regulation was identified as a remedy to address uncertainty regarding the accessibility of clinical supervisors across different jurisdictions, “*Regulation might then help this problem of clinical supervision … by creating a uniform standardized expectation of what that means and should look like” [P2].*

Accountability and enforceable mechanisms were viewed as a reflection of the extent to which practitioner conduct was monitored, and patients and clients could be protected from harmful practice and practitioners, *“...So if somebody complained…this person is regulated in Ontario, but helping somebody in Saskatchewan. Oh, well, that’s an unregulated province, so do we care?” [P8].* The regulatory patchwork for MHSUH services and providers was equated with the way governments and professions, as a reflection of society, care about client safety and quality services:

*I mean, we don’t for any other health issue…we don’t leave…big huge gaps and holes in the systems…We don’t do it for getting our teeth cleaned. Why are we doing it for addiction?… There needs to be enforceable standards…People are too vulnerable [P1].*

Some participants suggested that enforceable rigour, accountability to an oversight body (e.g., regulatory body), and harmonized standards would help prevent providers from slipping through the cracks.

#### Flexibility in regulatory approaches.

At the same time, participants expressed that harmonization of standards must include a degree of flexibility to account for different goals and desired autonomy of providers:

*The tricky part about a national framework is that not everybody has the same goals. And so, we’d have to be flexible enough to reflect that. And also, because of the criminalization history of substance use, there are a lot of individuals out there who do not want to be regulated and don’t want to have that power taken away from them again [P9].*

Concerns also emerged about the risk of over-regulation, especially in relation to some providers: *“Credentialism is a capitalistic sort of thing. And peer support is not intended to be rooted in capitalism” [P14].*

Participants agreed that no single model of regulation would be sufficient to accommodate the diverse landscape of MHSUH service provision across regions without simultaneously creating barriers to practice for some practitioner groups. Differences in geographic contexts, particularly in underserved communities, will likely require different regulatory models. Right-touch alternatives to statutory regulation such as certification and competency-based approaches, which match the strength of regulation with the level of risk to the public in a way that does not create unnecessary barriers to practice, were seen as a potential way to enhance access to a broader range of MHSUH providers.

#### Inclusivity for entry-to-practice.

Many participants discussed the importance of balancing consistency and inclusivity for entry-to-practice requirements. Most agreed on the need for clear standards and competency measures, regardless of the occupation they discussed. However, participants offered differing views on the nature of entry-to-practice standards. While adhering to traditional academic qualifications was identified by some to be an important part of being a unified, governable occupation, offering a lived experience pathway to demonstrate competencies was also acknowledged, *“…the competencies are at a master’s level, but you don’t necessarily need a master’s degree to reach a master’s level… I’m of the belief that master’s level competencies are what you need, and how you get them could vary” [P8].* Indeed, some participants asserted that to ensure safety and quality, we must abandon *“colonial era requirements for graduate training”* *[P11]* in favour of a more modern approach to measure who is competent to practice: *“the question is, can you do the job?...If you can do the job, you should be part of the profession” [P11]*.

These alternatives to traditional regulation were expressed as being most important for providers with lived and living experience: *“We wouldn’t want to do anything to dissuade or make more difficult, you know, entry into this for people with living experience” [P6]*. One proposed solution to overly strict entry-to-practice competencies included community-led quality assessment: *“involving the people receiving the care in the evaluation of the people delivering the care” [P6].*

### Supporting the integration of lived experience

#### Inequity in employment.

Participants expressed concern about workforce policies that constrain the pay, advancement, and overall integration into the workforce of MHSUH service providers who are not regulated by statute, many of whom draw on lived experience. One participant observed that *“there’s a bias and comfort with our government to continue to allocate money towards clinical services, as opposed to peer services” [P14].* Another described disparity in compensation across roles and workplace contexts, even for those who are performing the same role:

*Two people doing the exact same thing, like they could be outreach workers. And one could be a nurse who’s getting a higher salary, with benefits, with security, with the [regulatory] college to provide EAP and ongoing education and all those types of things. And there could be another outreach worker, that’s an individual that doesn’t have those qualifications, and therefore is making significantly less money, has less job security, and doesn’t have access to the same benefits [P9].*

Another participant identified that statutory regulation could raise wages so that peer support workers would be on par with those providers already regulated by statute, such as social workers, *“One of the benefits of regulation is the salary, the hourly rate will be higher” [P2].*

Speaking to growth and advancement among workers who approach service provision with a lived or living experience lens, one participant described how opportunities are limited for occupations that are not regulated by statute:

*Experience, which is ironically, exactly what you’re looking for, turns out to be the barrier that stops you from being able to get a job…the opportunities for upward growth, won’t necessarily go towards the peer support workers, but will go towards clinicians or other positions… it’s certainly a barrier to be able to maintain employment within a peer support field…there’s not enough thought about intentionally continuing to centre the lived experience perspective and these upper growth positions [P14].*

The imbalance between recruitment and retention of MHSUH service providers depending on regulatory status was considered particularly stark within hospital settings where differences in compensation and more formal or hierarchical team structures exacerbate challenges with integrating lived experience providers.

#### Wearing “both pant legs” of lived experience and statutory regulation.

Some participants cautiously suggested that statutory regulation may provide for stronger public protection, so long as it includes a distinct governance framework that acknowledges the unique expertise of individuals with lived or living experience. This was particularly the case regarding regulatory repercussions of self-disclosure, *“I’m told, as a clinician, never say that you have lived or living experience to your clients” [P7].* While many providers follow codes of ethics which discourage sharing personal experiences, the bedrock of peer support and lived experience work involves the application of wisdom acquired through life experiences that parallel those of their clients. Peer support and lived experience approaches can conflict with regulatory requirements:

*...if you’re supporting somebody and they’re at risk of suicide, you have to do whatever your clinical training tells you to do in that moment, not necessarily what your lived experience, intuition or knowledge tells you that you should do… so there’s just no way for a peer supporter to be a social worker, because they come in contradiction with each other [P14].*

Such statements reflect the challenges and dilemmas of wearing “*both pant legs*” *[P7]* in having lived experience and being a provider regulated by statute.

The criminalization of substances poses additional challenges for those who use lived and living experience approaches to provide services. One participant described how police background checks act as barriers to securing a role in their occupation as *“people with lived experience have experience with the criminal justice system” [P14].* Another noted the potential occupational risks of providers disclosing their lived experience using substances: *“Hidden peers exist across all populations… there are folks working with you who are hidden peers…we don’t ask them to come out in the light, so to speak, because they’re going to experience harms” [P7].*

#### Filling gaps by integrating lived experience approaches.

The perspective peer support workers offer was identified as one of several reasons to more fully integrate MHSUH workers with lived and living experience into systems and teams:

*Peers in their lived experience bring with them a difference of rationale, a difference of purpose, a difference of reason, and a different way of doing things...It is imperative at this point in the substance use workforce to have peers involved, if we don’t have them involved, and we don’t have them in the right way that [substance use health] continuum will collapse [P3].*

In addition to effectively involving them in the substance use health continuum, some participants expressed a preference to have peers as part of their support system, *“...I certainly would much rather someone with lived and living experience, who’s overcome the exact mental and behavioural disorders I have to support me with healing than someone who doesn’t have that lived and living experience…” [P6].* Participants who themselves were receiving care from a team acknowledged the value that professional clinical training provides while also identifying the potential for reciprocal learning between providers possessing and lacking lived and living experience on care teams.

Additional benefits of integrating lived experience providers included enhancing trust and connection when community members could fill paraprofessional roles to support outreach work. In Nunavut for example, *“these paraprofessional roles are essentially community members who may… have lived experience, but who are interested in…supporting community members and so…they have been able to increase capacity… in a way that is a bit more connecting and trusting” [P13].*

#### Power differentials between clinical and lived experience providers.

Participants described power differentials between clinical practitioners and workers with lived experience of MHSUH issues that can be highly stigmatized. Reciprocal collaboration, mutual respect, and co-creation in service delivery and policy development were identified as potential remedies for the power differentials they experienced in the workplace:

*We no longer want to be…viewed as…the things that we needed when we entered the system…, but rather seen from our expertise. That’s a complete flip, right? Co-creation looks a lot like allowing those classic power dynamic roles to be destabilized. [P7]*

Finally, many identified the need for stigma intervention and education, particularly for health care providers. Those with lived experience cited their own encounters of stigma from providers when seeking care and discussed a tendency to shape the presentation of their symptoms to obtain better care. As such, they suggested that data on MHSUH service use are consistently skewed, and this could be addressed if lived experience perspectives were at the centre of research, policy, education, and regulatory reform.

## Discussion

Our goal in this research was to gather perspectives from a range of subject matter experts on two focus areas: (1) how regulation and potential regulatory reform impact the safety and quality of services, access to services, and the workforce; and (2) how flexible, modern regulatory reforms in the MHSUH workforce can facilitate more equitable access to MHSUH services. Two themes were identified in relation to the first research question: impact of policy exclusion, criminalization, and stigma; and gaps in regulation limit access and public protection. These themes identify how stigma and a legacy of exclusion have created gaps in public protection and uneven access to care across jurisdictions. Two additional themes were identified in relation to our second research question: the dynamic tension between standardization and flexibility; and supporting the integration of lived experience.

While all participants agreed about the importance of quality assurance and expanding access, we found a dynamic tension between calls for standardization to better protect the public and calls for flexibility to better support the lived experience workforce. Gaps in statutory regulation contribute to gaps in access by constraining the pool of MHSUH service providers that meet eligibility criteria for employment-based benefits and Medicare coverage. Gaps in statutory regulation also contribute to gaps in quality assurance, leaving many people living in Canada with limited recourse for unsafe MHSUH care.

These gaps in regulation, access, and protection are themselves shaped by policy legacies. Without full inclusion under Canadian Medicare, MHSUH services and service providers have been neglected in terms of funding and regulation relative to physical health care. This policy neglect has been compounded by stigma around mental illness and substance use disorders, and by a long history of criminalization of substance use and some mental illnesses. The decentralized health system under Canadian federalism [67–69] has also contributed to confusing variations in regulatory frameworks and constrained accountability for the quality of MHSUH services. While the variability in MHSUH regulatory frameworks reflects these Canadian policy legacies, it also reflects fundamentally different views of the roles of regulatory structures and processes for different kinds of MHSUH services.

Other societal factors are at play beyond these policy legacies, particularly regarding the dynamic tension between standardization and flexibility. The distribution of power, autonomy, and risk also shapes values in health care [70] and contributes to gaps in regulation, access, and protection of service users and providers, particularly when integrating lived experience and expertise into the MHSUH workforce. Risk management strategies may “obscure the value of risk-taking activities” [71] (p. 104). Under this paradigm, the autonomy of people living with MHSUH challenges is seen as particularly problematic, as both their capacity and morality can be called into question: “People with mental health needs become designated either as good/responsible or deviant/irresponsible” [71] (p. 179).

There are signs of progress in addressing the gaps and policy legacies we identified in our research. Alberta and British Columbia advanced toward statutory regulation of counselling therapy and psychotherapy in 2024 [37]. Peer support is increasingly integrated into MHSUH services, including in Canada, the U.S., the U.K., and Australia [56,72–76]. Public funding supports addiction counsellors and harm reduction workers, though under different models [54,77]. Voluntary certification programs for peer support workers and addiction counsellors exist in Canada, along with competency frameworks for the substance use health and psychosocial rehabilitation workforces [38–42]. The legalization of cannabis since 2019 has begun to ease the burden of criminalization [78].

Looking ahead, opportunities include the potential for all provinces and territories to regulate psychotherapy and counselling therapy, with five provinces already doing so and two more in progress. Emerging paradigms in substance use health and structural stigma could challenge existing norms and support flexible regulation, particularly for the lived experience workforce [79,80]. The Stepped Care 2.0 model of MHSUH system transformation [81], which distributes risk across providers and agencies, and the concept of right-touch regulation, which aims to balance regulatory intervention with the level of risk, may guide future reforms so long as emphasis is placed on not creating undue barriers to practice. This approach aligns with recent recommendations from the World Health Organization to first understand the social and environmental context when introducing, evaluating, or updating regulatory frameworks for health practitioners [82].

The findings from this study suggest that right-touch regulation requires a different approach for peer support workers and other MHSUH service providers who draw primarily on lived experience. A lighter touch may not work since these MHSUH service providers are employed across the continuum of risk (from outreach and health promotion to in-patient hospital settings to crisis response, including suicide prevention) [83]. Here, the critical consideration shifts to minimizing the unique barriers to practice for this workforce, given their unique contribution, while supporting self-governed regulatory approaches such as voluntary certification and competency frameworks. Co-regulatory approaches are also possible, to the extent that funders set out certification and competency requirements for peer support workers, harm reduction workers, addiction counsellors, and psychosocial rehabilitation workers. For example, certified peer support workers are increasingly recognized as eligible Medicare providers by state governments in the U.S., with support from the federal government [84].

In our study, participants suggested more disruptive approaches to quality assurance that warrant exploration, including using competencies as entry-to-practice requirements and having service users play a leadership role in evaluating the quality of services received. Participants also pointed to the need for workforce policies to protect MHSUH providers from harm following disclosure of lived experience (including harms for ‘hidden peers’ in occupations such as nursing and medicine that are regulated by statute) [85–87] and to promote fair remuneration and career advancement opportunities for the lived experience workforce.

## Limitations

While this study captures a range of perspectives reflecting the broad and diverse MHSUH workforce landscape across Canada, these findings may not be transferable to MHSUH provider groups in different geographic, policy, and regulatory contexts. As this project focused on occupational groups that have limited or uneven access to statutory regulation, it did not include the full range of occupational and specialist groups that work to address MHSUH issues, particularly those with a longer history of statutory regulation (e.g., psychiatry, addictions medicine specialists, family physicians, nurses, psychology, occupational therapy and social work). Further, regulatory approaches for registered psychiatric nurses vary across the country in a manner similar to psychotherapy and counselling therapy, but were not included in the scope of this research [88]. Members of these other occupational groups may hold different perspectives on quality assurance.

Additionally, there is potential for researcher bias with the use of key informant interviews and our chosen participant selection and sampling strategies. Researcher bias may also come from three team members’ work as service providers and users in the MHSUH sector. Their unique experiences within their field and with regulation may have shaped their understanding of the data and its results. The potential for these biases was reduced through research team deliberations, advisory committee consultations, and rigorous analysis procedures. Moreover, thematic saturation was reached with our sample size [89]. Furthermore, while our use of qualitative description presents another limitation, the methodological flexibility it offered permitted us to explore issues related to regulation and quality assurance of the MHSUH workforce and describe what we found.

## Future research

Future research could focus on evaluating the outcomes of regulatory frameworks, expanding this area of research to include competency approaches for entry to practice, peer-led quality assurance (e.g., service users leading evaluation of services), and distributing risk among different system partners to achieve the ‘right touch’ in regulation. Comparative research might examine and contrast regulatory structures related to MHSUH workforces in different countries to highlight the contexts with the most promising regulatory approaches. To capture the full range of perspectives across the MHSUH landscape, a wider canvassing of MHSUH service providers not included in this study could be included in future studies.

## Conclusion

Regulation impacts the quality, availability and accessibility of the MHSUH workforce and the services it provides. Gaps in statutory regulation constrain the pool of MHSUH service providers that meet eligibility criteria for employment-based benefits and Medicare coverage and leave many Canadians with limited recourse for unsafe MHSUH care. Current regulatory frameworks reflect the impact of policy exclusion, criminalization of substances and stigma, and limited policies to support the integration of lived experience into the MHSUH and broader health workforce. Our findings indicate a need to re-imagine regulatory approaches for this workforce to accommodate the dynamic tension between standardization and public protection and support flexibility for lived experience providers and approaches to care. In addition to ensuring equitable access to MHSUH services and providers, re-imagined regulatory approaches are needed to address critical gaps in MHSUH workforce planning. There is potential for the concept of right-touch regulation to guide future reforms so long as not creating undue barriers to practice is emphasized along with matching regulation to the level of risk.

## Supporting information

S1 Table COREQ checklist. Consolidated criteria for reporting qualitative studies (COREQ): 32-item checklist.
(PDF)

S2 Table Semi-structured interview guide. (PDF)
