# Supplementary material for: Re-imagining approaches for mental health and substance use health workforce regulation in Canada: Making room for dynamic tensions
Source: PLOS Ment Health. 2025 Mar 10;2(3):e0000168. doi: 10.1371/journal.pmen.0000168 (PMC12798214; doi:10.1371/journal.pmen.0000168)
Supplement: S2 Table — (PDF) [file pmen.0000168.s002.pdf]

Supporting Information Table 2: Semi-Structured Interview Guide

Category	Guiding Questions	Potential Follow-Up Questions/Prompts
Introductory question	The MHSUH workforce includes a broad range of roles and services. Can you tell us briefly about your current role?	
Current regulatory or quality assurance mechanisms	*MHSUH providers in Canada use several models of regulation or quality assurance, including voluntary certification, competency frameworks, title protection, and statutory regulation.* Can you describe how regulation or quality assurance for services and providers is currently done in your field?<br><br>What factors do you think have influenced the development of this current framework?<br><br>To what extent do you think national uniformity is important in this area? Should there be harmonization or uniformity across Canada or is provincial variability acceptable/desirable?<br><br>Is there a role for further government action in this area? If so, what do you think should be the objectives of government action?	In your opinion, how effective are current regulatory or quality assurance models for MHSUH providers?<br><br>***Reform***: Are any alternative or new forms of regulation, registration, or certification being considered in your field?<br><br>***If voluntary mechanisms:*** What are some factors influencing members of your workforce choosing whether to complete these? Do you know if any data is collected on practice quality or safety for those who undertake these? What are the advantages and disadvantages of voluntary certification vs mandatory certification?<br><br>***Virtual****:* Were there any changes specific to virtual care delivery?

Impact of regulation on the public	*The main purpose of regulation is generally described as protecting the public, from unqualified, incompetent, or unethical providers.* You've already described the current situation for regulation and quality assurance for MHSUH providers in your area – could you expand on this in terms of public protection? So how is public protection currently achieved for MHSUH providers in your workforce area?<br><br>Are you aware of any workforce or regulatory policy changes (in Canada or internationally) that have been impacted access to MHSUH services among vulnerable populations?<br><br>Are there any changes you would like to see to workforce or regulatory policy to support safe and high quality MHSUH service provision to the public?	***Risks to the public:*** Do you think there are risks associated with MHSUH services provided by unregulated or otherwise unqualified providers? What factors have impacted this risk? (e.g., type of service provided, regulatory variability across Canada, workforce shortages, statutory regulation vs other approaches to quality assurance, changes during the pandemic?)<br><br>***If aware of workforce or regulatory policy that has impacted access to MHSUH services among vulnerable populations:*** How does gender, race, ethnicity, and other social categories factor into issues around access? How does cultural and linguistic diversity factor into access?<br><br>***Virtual****:* Were there any changes specific to virtual care delivery?
Impact of regulation on providers	*Regulatory and other quality assurance mechanisms can impact on MHSUH providers, including training, qualification, and recognition.* Can you describe how MHSUH providers in your workforce area currently enter practice or become trained as providers?<br><br>How do you think competency to practice can best be determined for MHSUH service providers in your workforce sector?<br><br>What role can/should lived experience play in MHSUH workforce regulation and quality assurance?<br><br>Are there any changes you would like to see to workforce or regulatory policy to support MHSUH providers?	Are any alternative or new forms of competence assessment or entry to practice requirements being considered in your field?<br><br>*Regulation and other quality assurance approaches are thought to potentially impact the status and acceptance of occupational groups within the health care system and interdisciplinary teams.* Do you think regulatory changes would impact the status or acceptance of MHSUH providers in this way? Is there a difference between the impact of statutory regulation vs certification or competency frameworks?

Impact of regulation on practice	*Regulatory and other quality assurance mechanisms can impact on practice standards, scopes of practice, and occupational titles.* Can you describe any impact that the current regulatory and other quality assurance structures have on service provision in your field?<br><br>What role do you think national or provincial standards play in ensuring safe, high-quality MHSUH services?<br><br>Should regulation or quality assurance of services or providers be changed or modernized in the current era of virtual service delivery?<br><br>Are there any changes you would like to see to workforce or regulatory policy to support MHSUH practice?	***National Standards*****:** A process to develop National Standards for Mental Health and Substance Use Services is currently underway in Canada. How could such standards support quality assurance for the MHSUH workforce? What would need to be included?<br><br>***Virtual MHSUH services:*** Are you aware of anything at the regulatory or quality assurance level that has either hindered or facilitated virtual MHSUH services? Are you aware of any challenges in offering cross-jurisdictional services virtually?<br><br>***Equity:*** Is there a role for regulation and other quality assurance mechanisms in addressing equity issues around virtual service provision? (e.g., underserved areas, low health literacy, marginalized populations?)
Magic wand or crystal ball	If you had a magic wand or a crystal ball, what would you want to change or to know to help ensure everyone in Canada had equitable access to safe and high-quality MHSUH services?	Are certain values inherent in defining safety/quality for specific sectors? (e.g., recovery-oriented, strengths-based, trauma-informed, culturally safe, sustainable)
Concluding thoughts	Is there anything else you would like us to know about regulation and quality assurance in the MHSUH workforce in Canada?	
